# Possible role of complement factor H in podocytes in clearing glomerular subendothelial immune complex deposits

**DOI:** 10.1038/s41598-019-44380-3

**Published:** 2019-05-27

**Authors:** Takeshi Zoshima, Satoshi Hara, Masakazu Yamagishi, Ira Pastan, Taiji Matsusaka, Mitsuhiro Kawano, Michio Nagata

**Affiliations:** 1 Division of Rheumatology, Department of Internal Medicine, Kanazawa University Graduate School of Medicine, Takara-machi 13-1, Kanazawa, Ishikawa 920-8640 Japan; 2 Kidney and Vascular Pathology, Faculty of Medicine, University of Tsukuba, 1-1-1, Ten-nodai, Tsukuba, Ibaraki 305-8577 Japan; 3 Department of Cardiovascular and Internal Medicine, Kanazawa University Graduate School of Medicine, Takara-machi 13-1, Kanazawa, Ishikawa 920-8640 Japan; 4 Laboratory of Molecular Biology, Center for Cancer Research, National Cancer Institute, NIH, 37 Convent Dr, Room 5106, Bethesda, Maryland 20892-4264 USA; 5 Department of Molecular Life Sciences, Tokai University School of Medicine, Shimokasuya 143, Isehara, Kanagawa 259-1193 Japan

**Keywords:** Lupus nephritis, Nephritis

## Abstract

Podocytes are known to express various complement factors including complement factor H (CFH) and to promote the removal of both subendothelial and subepithelial immune complex (IC) deposits. Using podocyte-selective injury model NEP25 mice and an IgG3-producing hybridoma clone 2B11.3 established by MRL/lpr mice, the present study investigated the role of podocyte complement regulation in only subendothelial IC deposition. In immunotoxin (LMB2) induced fatal podocyte injury (NEP25/LMB2) at day 12, glomerular CFH and C3a receptor (C3aR) expression was decreased as compared with NEP25/vehicle mice. In contrast, in sublytic podocyte injury 5 days after LMB2, glomerular CFH and C3aR expression was increased as compared with NEP25/vehicle mice. Intra-abdominal injection of 2B11.3 hybridoma to NEP25 mice (NEP25/hybridoma) caused IC deposition limited to the subendothelial area associated with unaltered CFH expression. NEP25/hybridoma mice with sublytic podocyte injury (NEP25/hybridoma/LMB2) resulted in increased glomerular CFH expression (1.7-fold) accompanied by decreased subendothelial IC deposition, as compared with NEP25/hybridoma. Immunostaining revealed that CFH was dominantly expressed in podocytes of NEP25/hybridoma/LMB2. In addition, puromycin-induced sublytic podocyte injury promoted CFH expression in immortalized mouse podocytes *in vitro*. These results suggest that in response to sublytic levels of injury, podocyte induced CFH expression locally and clearance of subendothelial IC deposits.

## Introduction

Glomerular immune complex (IC) deposition initiates glomerulonephritis via complement pathway activation and chemotaxis of inflammatory infiltrates^1^. The glomerular endothelial cell is the principal site of the endocapillary proliferative lesion induced by IC deposition. Actually, subendothelial IC is associated with membranoproliferative glomerulonephritis i.e., lupus nephritis, while subepithelial IC deposition as revealed by membranous nephropathy does not cause glomerular inflammatory influx. Thus, the local kinetics of subendothelial IC is of particular concern to elucidate the progression of glomerulonephritis.

The kinetics of glomerular ICs is largely dependent on the complement systems and inflammatory cells expressing Fc receptors. Complement components can contribute to the removal of ICs, concurrently with promotion of inflammation. C1q binds to IC and initiates classical pathway activation, leading to development of C3b. C3b covalently attaches to IC thereby promoting the solubilization of IC into the circulation^2^. The C3b-IC complex is bound by cells containing complement receptors 1 (CR1) such as human erythrocytes and rodent platelets, which are then transported to the phagocyte system^2^. The production of C3b further amplifies the solubilization of ICs through activation of the properdin system which is included in the alternative pathway^3^. In addition, complement regulatory factors that prevent injudicious complement activation could be also responsible for the clearance of ICs. Complement factor H (CFH), a representative complement regulatory component, acts as CR1 on rodent platelets^4^, and systemic CFH depletion has been shown to increase glomerular ICs in rodent IC-mediated glomerulonephritis models^5^. In this way, complement regulatory factors are one of the key players in the degradation of IC deposition.

Podocytes maintain the glomerular environment locally by expressing various factors in both normal and diseased states^6,7^. Podocytes also express complement regulatory factors *in vivo* and *in vitro*, suggesting that they regulate complement at this level. Podocyte cell line has been demonstrated to expresses various complement factors, including C1q, C1r, C2, C3, C3a receptor (C3aR), C5a receptor (C5aR), C7, CR1-related gene/protein y (Crry), decay-accelerating factor (DAF), complement factor I (CFI) and CFH^8^. The expression of these complement factors is affected by a variety of stimuli that injure podocytes^8,9^. Especially, CFH has been shown to be produced in podocytes in *in vivo* studies^9–11^, with its expression induced by functional changes of podocytes in the sublytic injury setting^9^. In the mouse model of IC-mediated glomerulonephritis induced by chronic serum sickness (CSS), podocytes expressed CFH and facilitated the removal of glomerular ICs in both the subepithelial and subendothelial areas, and seemed to be the functional surrogate for human CR1^10^. However, whether only subendothelial IC deposition promotes the expression of complement regulatory components on podocytes and processes the subendothelial IC remains to be determined. This uncertainty prompted us to investigate the impact of sublytic podocyte injury on the regulation of subendothelial IC deposition.

## Results

### Podocyte loss caused complement regulatory factor inhibition in the glomeruli

**Figure 1:**
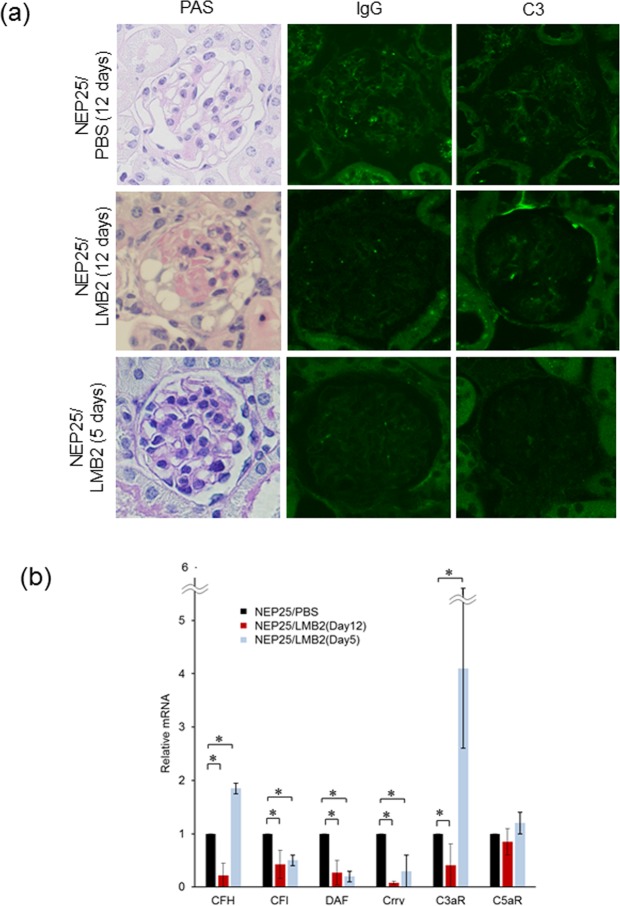
Podocyte loss downregulates complement regulatory factors. (**a**) NEP25/LMB2 mice (12 days after LMB2 exposure, n = 3) showed fibrin deposition and epithelial cell hyperplasia without IC deposition. Magnification, x400. (**b**) qRT-PCR analysis of isolated glomeruli showed that podocyte loss induced a decrease in complement regulating factor mRNAs. NEP25/PBS (n = 3), NEP25/LMB2 (Day 12, n = 3), NEP25/LMB2 (Day 5, n = 3). *p < 0.05. CFH; Complement factor H, CFI; complement factor I, DAF; decay-accelerating factor, Crry; complement receptor 1-related gene/protein y, C3aR; C3a receptor, C5aR; C5a receptor.

Podocyte-specific injury model NEP25 mice were injected once with immunotoxin (LMB2) or phosphate buffered saline (PBS), the latter serving as controls. Twelve days later, histopathological evaluation was conducted in both groups. As previously reported^12^, LMB2-treated NEP25 mice (NEP25/LMB2) at 12 days showed glomerular tuft collapse with epithelial cell hyperplasia accompanied by extensive podocyte loss, resembling collapsing focal segmental glomerulosclerosis (FSGS) (Fig. 1a). Such findings were absent in PBS-treated controls (NEP25/PBS). NEP25 mice with and without LMB2 did not show any IgG or C3 deposition in glomeruli.

qRT-PCR analysis revealed that mRNA expression of complement regulatory factors such as CFH, CFI, DAF, Crry, and C3aR was significantly decreased in the isolated glomeruli of NEP25/LMB2 at 12 days compared to NEP25/PBS (Fig. 1b). Immunostaining revealed that glomerular C3aR was expressed at podocytes, with its expression decreased in NEP25/LMB2 compared with NEP25/PBS (Supplemental Fig. 1). These results suggest that podocyte loss resulted in inhibition of complement regulatory factor production in glomeruli.

### Sublytic podocyte injury attenuated IC deposition in the glomerular subendothelial area

**Figure 2:**
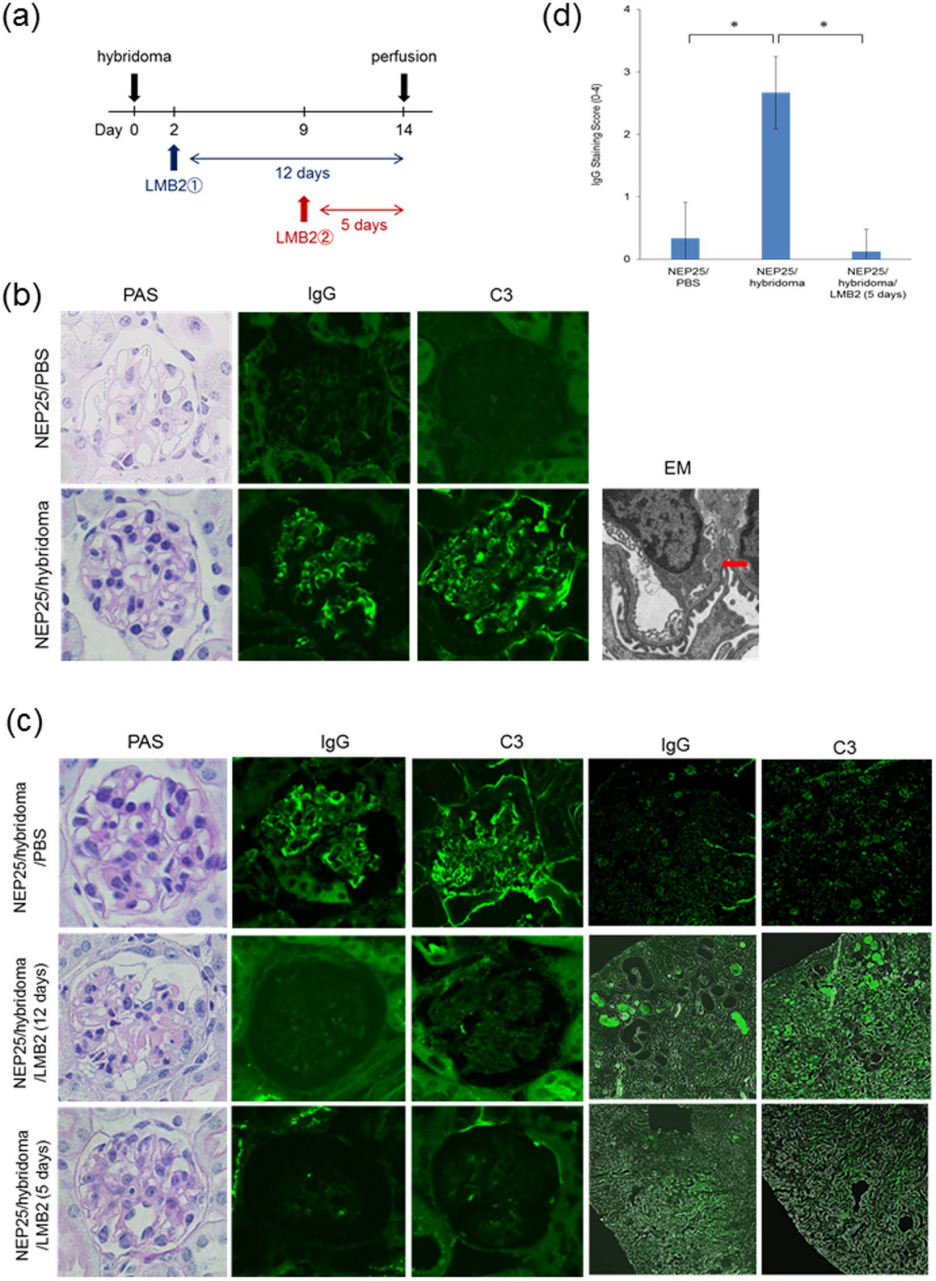
Sublytic injured podocytes attenuate immune complex deposition in subendothelial area *in vivo*. (**a**) Experimental schedule. LMB2① means 12 days after LMB2 exposure. LMB2② means 5 days after LMB2 exposure. (**b**) NEP25/hybridoma/PBS mice (n = 3) showed no abnormality in light microscopic study, while IgG and C3 deposits were observed along with capillary wall in immunofluorescence. Magnification, x400. Electron microscopy (EM) showed electron dense deposition only in the subendothelial area (arrow). Magnification, x700. (**c**) NEP25/hybridoma/LMB2 mice (12 days after LMB2 exposure, n = 3) showed fibrin deposition and epithelial cell hyperplasia without endocapillary proliferative lesions, similar to the findings in NEP25/LMB2 mice. Both IgG and C3 deposits were scant in the glomeruli, but were concentrated in the tubular lumen. NEP25/hybridoma/LMB2 (5 days after LMB2 exposure, n = 8) showed minor glomerular abnormalities without IC deposition in either glomeruli or tubular lumen. Magnification, x400 in the picture of glomerulus, or x100 in the picture of renal parenchyma. (**d**) Staining intensity score for IgG in glomeruli. NEP25/hybridoma/PBS mice showed significant increase of IgG deposition, whereas LMB2 treatment reduced the deposition in NEP25/hybridoma/LMB2 mice. NEP25/PBS (n = 3), NEP25/hybridoma/PBS (n = 3), and NEP25/hybridoma/LMB2 (5 days after LMB2 exposure, n = 8). *p < 0.05.

We speculated that injured podocytes would regulate complement expression and glomerular IC deposition. We tested whether hybridoma-derived glomerular IC deposits would be altered by podocyte injury in the NEP25/LMB2 (Fig. 2a). In PBS-treated controls, MRL/lpr mice-derived hybridoma, clone 2B11.3, used in this study caused IgG and C3 deposition along the capillary wall as determined by immunofluorescence at 14 days after hybridoma injection, despite the absence of any apparent features of proliferative glomerulonephritis (NEP25/hybridoma/PBS) (Fig. 2b). Electron microscopy showed electron dense deposition only in the subendothelial area (Fig. 2b). Hybridoma and LMB2-treated NEP25 mice (NEP25/hybridoma/LMB2) at 12 days showed collapsing FSGS lesions similar to those in the NEP25/LMB2 without hybridoma treatment (Figs 2c and 1a). Immunofluorescence showed no IgG or C3 deposition in the glomeruli, while accumulation of IgG and C3 was found in tubular lumens of NEP25/hybridoma/LMB2 at 12 days, as compared to NEP25/hybridoma/PBS (Fig. 2c). These results suggest that podocyte loss caused subendothelial IC leakage to tubular lumens.

To clarify whether disruption of the glomerular filtration barrier by podocyte loss alone is the cause of IC leakage to the urine or whether podocytes directly remove subendothelial IC, we induced sublytic podocyte injury at 5 days after LMB2 injection (Fig. 2a), wherein podocytes remain attached to the glomerular basement membrane (GBM) in the prior state of detachment (Supplemental Fig. 2). Sublytic podocyte injury of NEP25/hybridoma/LMB2 at 5 days showed intact glomeruli without FSGS lesion (Fig. 2c). Notably, glomerular IgG and C3 deposits were significantly reduced in NEP25/hybridoma/LMB2 at 5 days as compared to NEP25/hybridoma/PBS, without any positivity in tubular lumen (Fig. 2c and d), suggesting that sublytic podocyte injury promoted the clearance or inhibition of subendothelial IC deposition without leakage to tubular lumens.

### Sublytic podocyte injury increased glomerular complement factor H expression

To determine what factor associates with IC decrease in sublytic podocyte injury, qRT-PCR was performed using isolated glomeruli.

**Figure 3:**
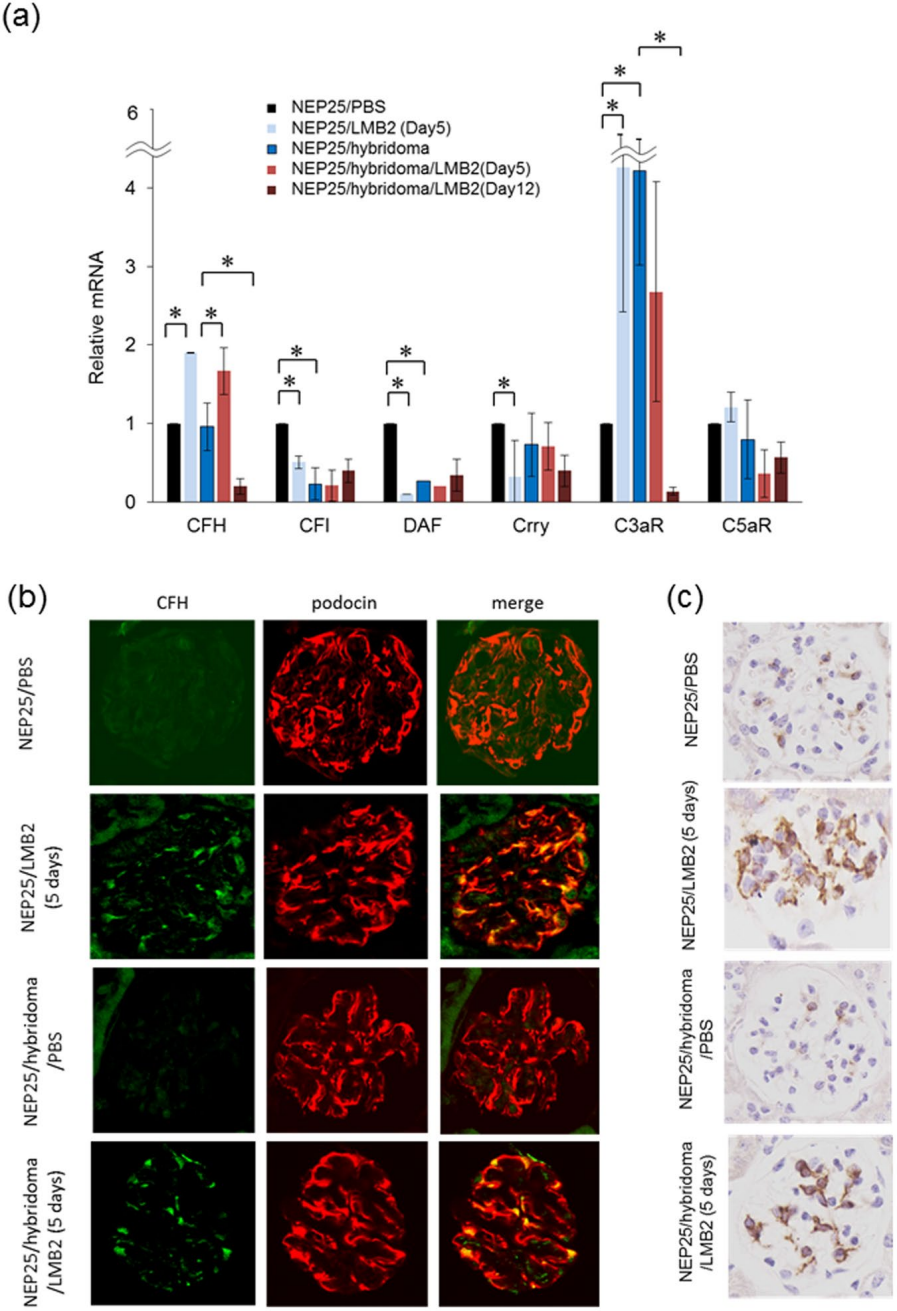
Sublytic podocyte injury induces CFH *in vivo*. (**a**) qRT-PCR analysis of isolated glomeruli showed increased CFH expression in the setting of sublytic podocyte injury both with and without hybridoma 5 days after LMB2 exposure. The mRNA expression was compared in NEP25/PBS vs. NEP25/LMB2 (Day 5), NEP25/PBS vs. NEP25/hybridoma/PBS, NEP25/hybridoma/PBS vs. NEP25/hybridoma/LMB2 (Day 5), and NEP25/hybridoma/PBS vs NEP25/hybridoma/LMB2 (Day 12) mice. NEP25/PBS (n = 3), NEP25/LMB2 (Day 5, n = 3), NEP25/hybridoma/PBS (n = 3), NEP25/hybridoma/LMB2 (Day 5, n = 8), NEP25/hybridoma/LMB2 (Day 12, n = 3). *p < 0.05. (**b**) Immunofluorescence of CFH and podocin (podocyte marker) showed increased podocyte-derived CFH in the setting of sublytic podocyte injury. Magnification, x400. (**c**) Immunohistochemistry showed increased CFH staining in the position of podocyte in the setting of sublytic podocyte injury. Magnification, x400.

First, sublytic podocyte injury in NEP25/LMB2 at 5 days increased CFH and C3aR mRNA, and decreased CFI, DAF and Crry mRNA compared to NEP25/PBS controls (Fig. 3). Of particular note, CFH and C3aR expression in sublytic podocyte injury was quite different from that accompanying podocyte loss in NEP25/LMB2 at 12 days, namely decreases of these components (Fig. 1b). Next, NEP25/hybridoma/PBS showed increased C3aR mRNA, and decreased CFI and DAF mRNAs as compared with NEP25/PBS controls, even though CFH mRNA was unaltered. This means that subendothelial IC deposition alone is not sufficient to increase glomerular CFH expression. Finally, in NEP25/hybridoma/LMB2 at 5 days, CFH mRNA increased significantly (1.7-fold) while the mRNA of other complement regulatory factors did not, as compared to NEP25/hybridoma/PBS.

To determine which glomerular cells predominantly express CFH in the podocyte sublytic state, we performed CFH immunostaining *in vivo*. Immunofluorescence showed overt expression of CFH in the glomeruli of NEP25/hybridoma/LMB2 and NEP25/LMB2 at 5 days, but not in those of NEP25/hybridoma/PBS at 12 days or NEP25/PBS (Fig. 3b). CFH was predominantly co-localized with podocin (podocyte marker), although other glomerular areas also showed only partial CFH expression. Immunohistochemistry confirmed CFH expression in the glomeruli in NEP25/hybridoma/LMB2 and NEP25/LMB2 at 5 days as the results of immunofluorescence (Fig. 3c).

To clarify whether circulating CFH could affect the kinetics of subendothelial IC deposits, serum CFH was evaluated. Circulating CFH level showed no significant difference between NEP25/hybridoma/PBS and NEP25/hybridoma/LMB2 at 5 days (0.48 ± 0.10 vs 0.41 ± 0.08 optical density units; p = 0.48).

These data suggest that sublytic podocyte injury results in increased glomerular CFH expression predominantly in the podocytes without serum CFH elevation, leading to decreased subendothelial IC deposition.

### Puromycin-induced sublytic injury in podocytes induced CFH expression *in vitro*

**Figure 4:**
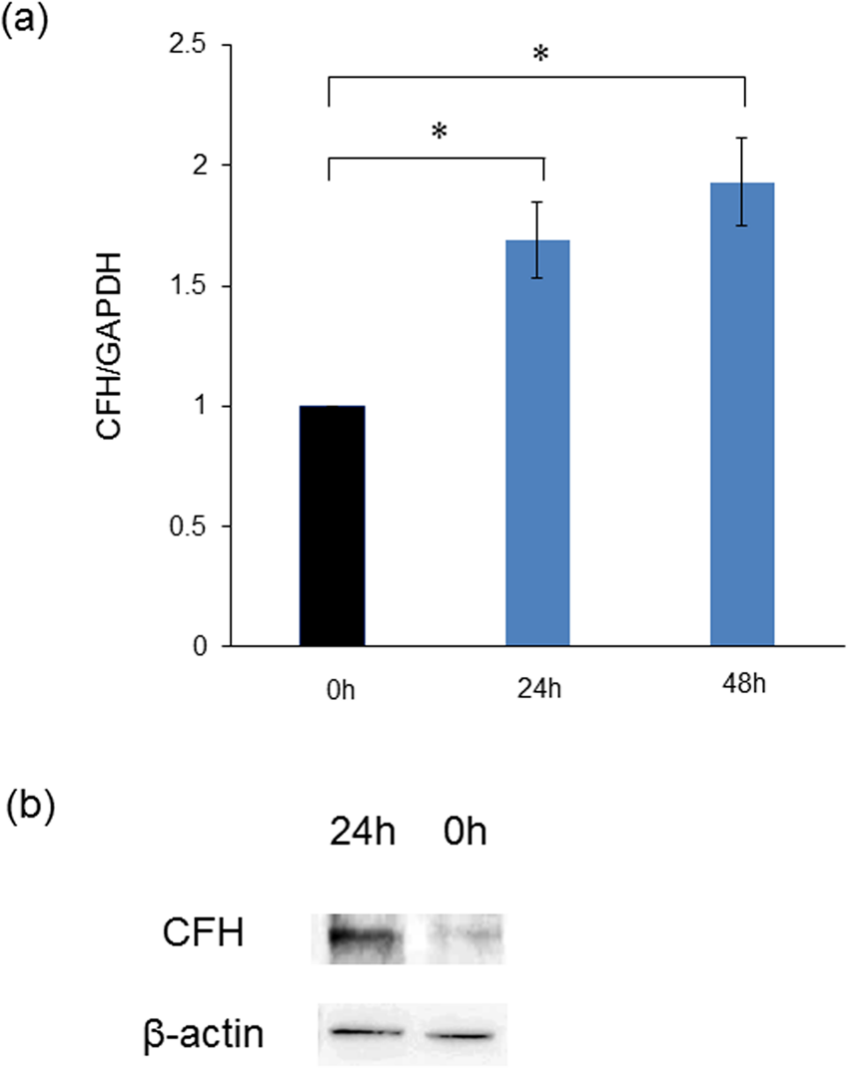
Sublytic podocyte injury induces CFH *in vitro*. (**a**) CFH mRNA expression was increased by puromycin-treated mouse immortalized podocytes *in vitro*. *p < 0.01. (**b**) Western blotting showed increased CFH protein in PAN-treated podocytes for 24 hours as compared to controls. The full-length gels are presented in Supplementary Fig. 4.

To examine whether podocytes increase CFH expression in the setting of sublytic injury, qRT-PCR was performed using immortalized mouse podocytes treated with puromycin aminonucleoside (PAN). The concentration and period of PAN treatment seemed to be sufficient to induce sublytic podocyte injury^13^. CFH mRNA expression increased significantly at both 24 and 48 hours after the treatment with PAN (Fig. 4). PAN-treated podocytes also increased C3aR mRNA expression but did not alter the expression of other complement regulatory factors such as CFI, DAF, Crry or C5aR (Supplemental Fig. 3), similar to the findings of glomerular mRNA expression of NEP25/ LMB2 (day5) in an *in vivo* study. Western blotting confirmed increased CFH protein in PAN-treated podocytes for 24 hours as compared to controls (Fig. 4b). These results highlight the induction of CFH by sublytic podocyte injury.

## Discussion

The present study focused on the role of intrinsic glomerular cell-derived complement factors as a local processor of subendothelial IC deposition. Previous studies using a CSS model of IC-mediated glomerulonephritis concluded that podocyte-derived CFH was involved in the processing of both subendothelial and subepithelial IC deposits^5,10^. The present study instead employed MRL/lpr mice-derived hybridoma clone 2B11.3 that caused IC deposition limited to the subendothelial area without any features of glomerulonephritis. In addition, in a podocyte specific injury model, NEP25 mice, the role of podocyte-derived complement factors was focused on. Both provided an appropriate environment to examine the role of podocytes in only subendothelial IC deposition *in vivo*.

Our first finding is that podocyte loss at 12 days of LMB2 treatment promoted IC leakage from the glomeruli to tubular lumens, whereas sublytic podocyte injury at 5 days was associated with no IC deposition in the glomeruli or tubular lumina. This suggests that sublytic injury in podocytes facilitates removal of subendothelial IC deposits synthesized by hybridoma or inhibits IC formation.

Next we analyzed the profiles of complement regulatory factors in podocyte loss at 12 days and sublytic podocyte injury at 5 days. The majority of complement regulatory factors mRNA in isolated glomeruli in podocyte loss showed a significant decrease, whereas sublytic injury in podocytes showed increases of CFH and C3aR mRNA. The increase of CFH mRNA was supported by the finding of PAN-induced sublytic injury in podocytes *in vitro*. NEP25/hybridoma/PBS showed subendothelial IC deposition and no change in glomerular CFH mRNA, in contrast to NEP25/hybridoma/LMB2 with sublytic podocyte injury at 5 days which revealed attenuated subendothelial IC deposition and increasing glomerular CFH mRNA. Notably, immunostaining showed that glomerular CFH expression was predominantly located at podocytes, although only a partial expression was observed in other glomerular areas *in vivo*. In addition, PAN-induced sublytic injury in podocytes promoted CFN mRNA and protein expression *in vitro*. These results suggest that podocytes locally regulate expression of complement regulatory factors, particularly up-regulation of CFH and remove subendothelial IC deposits against a background of sublytic injury. The increase of C3aR mRNA in a sublytic podocyte injury model might reflect a response against podocyte injury factors as suggested by a previous *in vitro* and *in vivo* study^8^, although the role of C3aR remains undetermined in IC-mediated glomerulonephritis^14,15^.

The mechanism whereby sublytic injury in podocytes, representing neither a healthy state nor detachment which is fatal, causes increased CFH is unknown. Previously, it was shown that injured podocytes induce CFH in a rodent membranous nephropathy model^9^. In passive Heymann nephritis caused by anti-Fx1A antibody, complement was activated on podocytes associated with increased CFH expression^9^. In addition, cultured podocytes in a condition of complement activation induced by anti-Fx1A antibody-driven sublytic injury increased CFH over time *in vitro*. These findings suggest that subepithelial IC deposits in membranous nephropathy stimulated complement activation and CFH expression in podocytes. The present study revealed in contrast that subendothelial IC deposition by hybridoma injection per se did not alter glomerular CFH mRNA expression, whereas the sublytic podocyte injury induced in PBS- or hybridoma-injected mice increased CFH mRNA expression, indicating that sublytic injury per se stimulates CFH in podocytes.

The function of sublytic podocyte injury-driven CFH may be local processing of subendothelial IC deposition. Alexander *et al*. demonstrated that glomerular deposition of IgG and C3 in glomeruli was increased in CFH deficient mice in a CSS model, as compared with wild type (WT) controls with CSS^5^. Likewise, WT mice with CFH deficient kidneys exhibited greater CSS-induced IC deposition in the subendothelial and subepithelial areas, as compared to CFH deficient mice with WT kidneys^10^. These results suggest that the podocyte-derived CFH, rather than circulating CFH, is important for the processing of both subendothelial and subepithelial IC deposition and that CFH in rodent podocytes seems to be the functional surrogate of human CR1^10^. Similarly, CFH deficient MRL/lpr mice showed increased IC deposition in the subendothelial and subepithelial areas, as compared with CFH sufficient MRL/lpr mice^16^. Our model also showed no difference in serum CFH levels between NEP25/hybridoma/PBS and NEP25/hybridoma/LMB2. These results highlight the important role of CFH in local processing of IC deposition in both the subendothelial and subepithelial areas, rather than the effect of circulating CFH, suggesting that podocyte-derived CFH facilitates the local adaptation needed to remove subendothelial IC deposits.

Several limitations may have affected the validity of the present study. First, the direct effect of podocyte-derived CFH in removing IC was not addressed. Inhibition of podocyte-derived CFH in this model would be our next step. Second, the mechanism of podocyte-derived CFH in removing IC remains undetermined, including (1) whether CFH can pass through GBM, (2) whether CFH is related to formation of complex with deposited IC, (3) how the effect of CFH on C5b-9 and CD59a is manifested in relation to subendothelial ICs. Complement system is necessary in translocation of subendothelial IC across GBM by solubilization of IC^17,18^. In addition, although CFH may be related to the formation of complexes between CFH and IC because CFH can play the same role of complement receptor 1 by combining with C3b-IC complex^4^, how podocyte-derived CFH passes through GBM and attaches to subendothelial ICs remains undetermined because CFH is around 150 kDa which would preclude its translocating GBM due to its size barrier^19^. Furthermore, CD59a is also expressed in the glomeruli, although which cell-types express CD59a has not been specified^20^. Third, weak expression of CFH was also observed in the glomerular tuft, in addition to podocytes. This means that CFH expression in other glomerular resident cells may not have been fully excluded, although their contribution would seem to be relatively small. Various stimuli affect mesangial cells to express CFH^9,21–23^. Glomerular endothelial cells also could express complement regulatory factors in a VEGF-treated condition^11^. Fourth, the mechanism of CFH expression in sublytic injury in podocytes was not determined. Finally, a similar serum level of CFH may not be sufficient to exclude the effect of liver-produced circulating CFH in this model, suggesting the possibility that both podocytes- and circulation-derived CFH exert an impact on the clearance of IC in our model. In this way further studies will be needed to more precisely characterize the role of podocytes in subendothelial ICs.

In conclusion, sublytic injury in podocytes induces glomerular CFH expression, and may serve to remove IC deposits from the glomerular subendothelial area. Our result is novel and of importance in drawing attention to a new role of podocytes in the local pathophysiology of IC-mediated glomerulonephritis.

## Materials and Methods

### Animal study

#### NEP25 mice

The FSGS model is a previously established mouse model genetically expressing human CD25 limited to podocytes (NEP25 mouse)^12^. The single injection of human CD25-specific immunotoxin (LMB2) can induce selective injury in podocytes and cause severe proteinuria and histological characteristics of FSGS. Mice were maintained at the animal facility of the Institutional Animal Use and Care Committee at the University of Tsukuba (registration no. 16-234) in accordance with its institutional guidelines. The 8- to 12-week-old mice used for the experiments were monitored while having free access to water and standard mice chow. NEP25 mice were injected with LMB2 (2.5 ng/g body weight dissolved in 0.1 ml of PBS containing 0.1% bovine serum albumin) or PBS through the tail vein. After 5 or 12 days, the mice were euthanized and kidney tissues were collected. All experiments in the present study were approved by the Institutional Animal Use and Care Committee at the University of Tsukuba (registration no. 16-234).

#### Hybridoma

A nephritogenic IgG3 antibody-producing hybridoma clone, 2B11.3, derived from an unmanipulated MRL/lpr mouse was used in this study. When injected into severe combined immunodeficiency mice, this hybridoma induced endocapillary proliferative glomerulonephritis^24^. For this study, this hybridoma (1 × 10^7^ cells) was injected intraperitoneally into NEP25 mice aged between 8 and 12 weeks. Mice were euthanized 14 days after the injection and the kidneys were obtained for histopathological examination.

#### Histostaining and morphological analyses

All mice were anesthetized with 30% isoflurane and perfused with 4% paraformaldehyde (Wako, Osaka, Japan). After the kidneys were obtained, renal tissues were fixed in 4% paraformaldehyde and embedded in paraffin for light microscopic study. Paraffin sections (2 μm) were processed for periodic acid-Schiff (PAS) as described elsewhere^25^. Parts of the kidney samples were incubated in graded (10–20%) sucrose-PBS solution and frozen for immunofluorescence study or were fixed with 2% glutaraldehyde for electron microscopy study as described elsewhere^26^. For immunofluorescence analysis, primary antibodies to mouse C3 (Hycult Biotechnology, Uden, the Netherlands), mouse CFH (Hycult Biotechnology, Uden, the Netherlands) and mouse podocin (Sigma Aldrich, St. Louis, MO, USA) were reacted with secondary antibodies labeled with Alexa 488 or Alexa 568 (Life Technologies, Carlsbad, CA). FITC-labeled anti-mouse IgG antibodies (Cappel, Organon Technika NV, Turnhout, Belgium) were used directly. Semiquantitative scoring of IgG staining intensity from 0 to 4 was conducted in 10 glomeruli from each mouse as reported previously^27^. Immunofluorescence images were obtained using a confocal laser scanning microscope.

### Glomerular isolation

Glomerular isolation was carried out as described elsewhere^28^ with a slight modification. In brief, mice were anesthetized and perfused with PBS thoroughly to rid the glomeruli of all blood constituents including plasma and platelet-associated CFH^9^, followed by PBS containing 1 mg/ml of ion powder. After perfusion, the kidneys were removed and cut into 1-mm3 pieces and digested in collagenase A (Roche, Basel, Switzerland) at 37 °C for 30 minutes with gentle shaking. The tissue was pressed gently through a 100-μm cell strainer, and glomeruli containing iron powder were then gathered using a magnetic particle concentrator. An aliquot (1:1500) of the glomerular isolate was visualized under a microscope to ensure that the sample contained fewer than 5 tubular fragments per 3200 field. Most isolated glomeruli (80%) were decapsulated, similar to what had been reported previously^28^. During the procedure, kidney tissues were kept at 4 °C except for the collagenase digestion at 37 °C.

### Total RNA extraction and quantitative real time- PCR (qRT-PCR) analysis

Total RNA of isolated glomeruli or immortalized podocytes was extracted using ISOGEN (Wako Pure Chemical Industries, Osaka, Japan) as described elsewhere^26^. RNA was quantified using a Nanodrop 1000 spectrophotometer (Thermo Scientific, Rockford, IL). Total RNA (1 μg) was reverse transcribed using the Thermoscript RT-PCR System (Life Technologies, Tokyo, Japan) for first-strand cDNA. Subsequently, 10 ng of cDNA template and 0.25 mmol/L of sequence-specific primers (listed in Supplementary Table) were used for qRT-PCR with a KAPA SYBR Fast qPCR Kit (Nippon Genetics, Tokyo, Japan) and an ABI 7300 real time PCR system (Life Technologies, Tokyo, Japan) as described elsewhere^29^. All of the measured values were normalized with glyceraldehyde-3-phosphate dehydrogenase (GAPDH) or 18 S ribosomal RNA, and calculated according to the ΔΔCT method.

### *In vitro* study

An immortalized mouse podocyte cell line (passages 10–16) was used^30^. As described elsewhere^18^, on collagen type I- (Koken, Tokyo, Japan) coated dishes, podocytes were cultured in RPMI-1640 medium (Biochrom, Berlin, Germany), containing heated 10% fetal bovine serum (PAA, Pasching, Austria), 100 U/ml penicillin, and 100 mg/ml streptomycin (Sigma-Aldrich, St Louis, MO, USA). Podocytes were expanded by culture in medium containing 10 U/ml mouse interferon-γ (Sigma-Aldrich, St Louis, MO, USA) at 33 °C. Removal of interferon-γ and switching the growth temperature to 37 °C for 10–14 days caused podocytes to stop proliferation and assume a differentiated phenotype^31^. Podocyte injury was induced by treatment with 100 μg/ml PAN for 24–48 hours. The cells were also subjected to total RNA or protein.

### Western blotting

Proteins from immortalized mouse podocyte cells were extracted and solubilized in radioimmunoprecipitation assay buffer with protease inhibitors. Proteins were separated by 7.5% polyacrylamide gel electrophoresis (SDS-PAGE) and transferred to polyvinylidene difluoride membranes (Bio-Rad, California, USA). Blots were incubated with polyclonal mouse anti-mouse CFH (Hycult Biotechnology, Uden, the Netherlands). After washing in blocking solution, primary antibodies were detected using a horseradish peroxidase-conjugated antibody (GE Healthcare Japan) and a chemiluminescent substrate (Thermo Scientific, Rockford, IL).

### Measurement of serum CFH

Serum CFH levels was analyzed by enzyme-linked immunosorbent assay (ELISA) by using goat antibodies (Quidel, California, USA). The amounts were expressed by the value of absorbance at optical density units at 450 nm because of a lack of high quality purified CFH protein for use as the standard.

### Statistics

The results are expressed as means ± SE. Statistical significance was determined with a two-tailed Student’s t-test or Mann-Whitney’s U test as appropriate. In all calculations, the significance of differences was accepted when the P value was < 0.05.

All data generated or analyzed during this study are included in this article and the supplemental files.

## Supplementary information

Supplementary informatioin
