# Supplementary material for: Possible role of complement factor H in podocytes in clearing glomerular subendothelial immune complex deposits
Source: Sci Rep. 2019 May 27;9:7857. doi: 10.1038/s41598-019-44380-3 (PMC6536504; doi:10.1038/s41598-019-44380-3)
Supplement: Supplementary file 1 — Supplementary informatioin [file 41598_2019_44380_MOESM1_ESM.docx]

Supplementary information

Possible role of complement factor H in podocytes to in clearing glomerular subendothelial immune complex deposits

Takeshi Zoshima^1), 2)^, Satoshi Hara^1)^*, Masakazu Yamagishi^3)^, Ira Pastan^4)^, Taiji Matsusaka^5)^, Mitsuhiro Kawano^1)^, and Michio Nagata^2)^

1) Division of Rheumatology, Department of Internal Medicine, Kanazawa University Graduate School of Medicine, Takara-machi 13-1, Kanazawa, Ishikawa 920-8640, Japan

2) Kidney and Vascular Pathology, Faculty of Medicine, University of Tsukuba, 1-1-1, Ten-nodai, Tsukuba, Ibaraki, 305-8577, Japan.

3) Department of Cardiovascular and Internal Medicine, Kanazawa University Graduate School of Medicine, Takara-machi 13-1, Kanazawa, Ishikawa 920-8640, Japan

4) Laboratory of Molecular Biology, Center for Cancer Research, National Cancer Institute, NIH, 37 Convent Dr, Room 5106, Bethesda, Maryland, 20892-4264, USA.

5) Department of Molecular Life Sciences, Tokai University School of Medicine, Shimokasuya 143, Isehara, Kanagawa 259-1193, Japan

Supplemental Figure 1


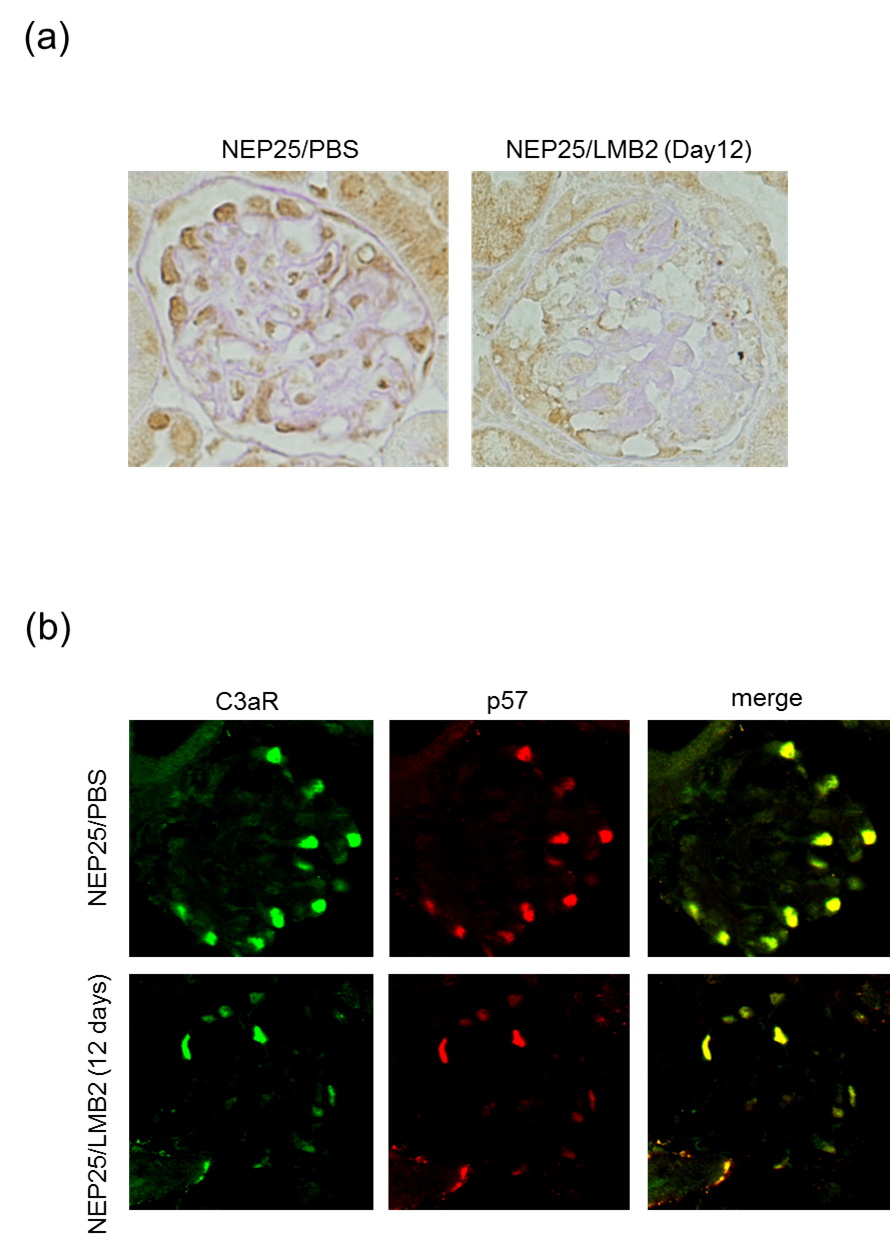


(a) NEP25/LMB2 mice (12 days after LMB2 exposure) showed reduced C3aR staining in the glomeruli compared to NEP25/PBS mice by immunohistochemistry. Magnification, x400.

(b) Immunofluorescence of C3aR and p57 (podocyte marker) showed reduced staining in the setting of podocyte loss. Magnification, x400.

Supplemental Figure 2


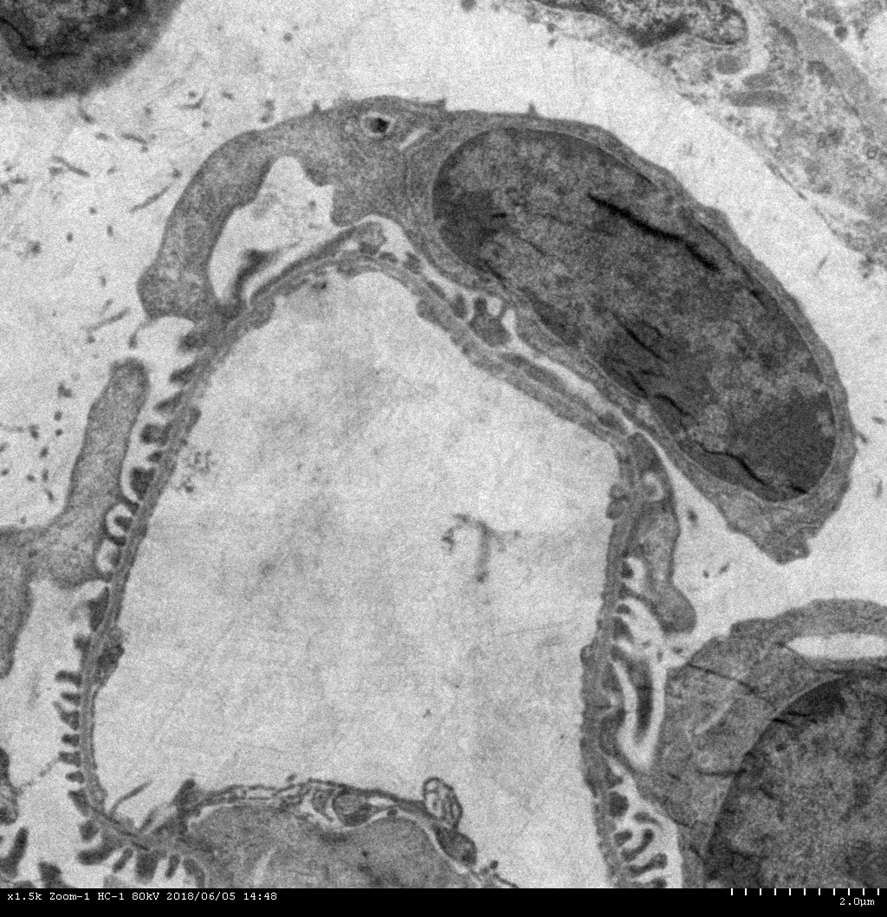


Electron microscopic features of NEP25/LMB2 mice (5 days after LMB2 exposure) with sublytic injury. Podocyte cytoskeletons are substantially intact with partial foot process effacement. Magnification, x700.

Supplemental Figure 3


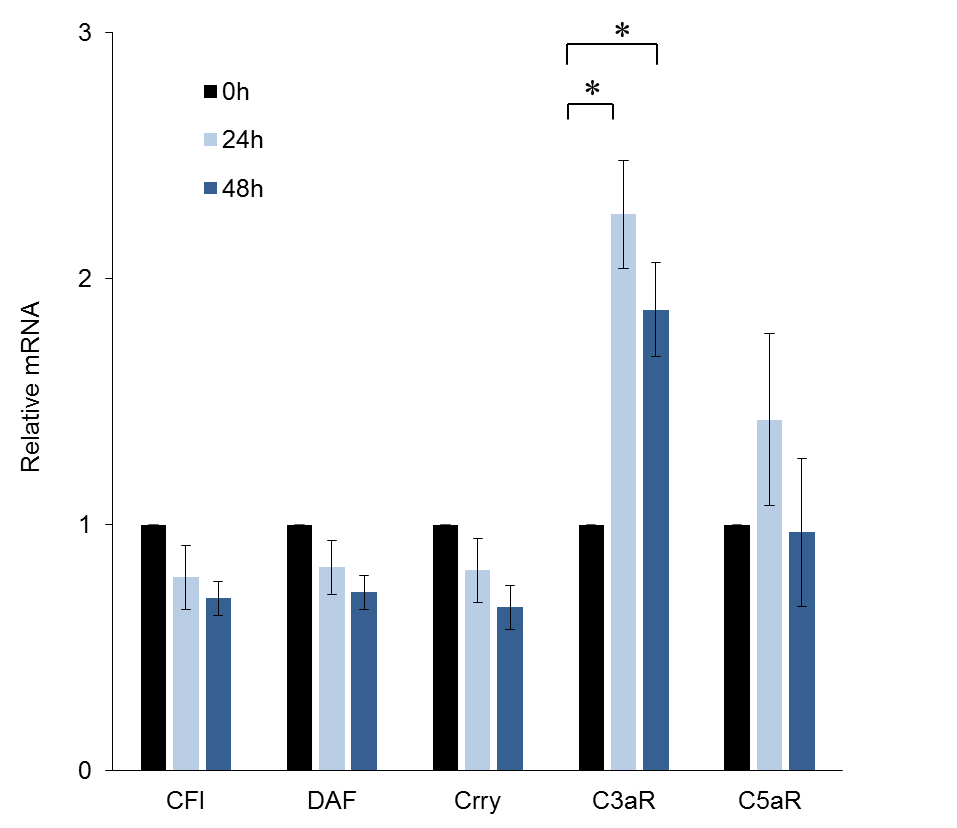


qRT-PCR analysis of podocytes showed the similarity of expression of other complement regulatory factors between in vitro and in vivo in the setting of sublytic podocyte injury. *p<0.05.

Supplemental Figure 4

Uncropped Western blots


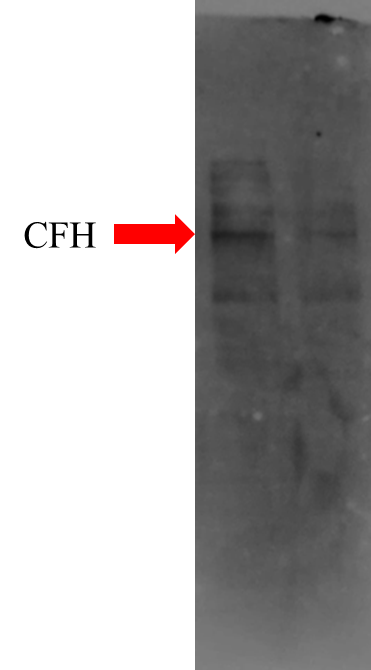


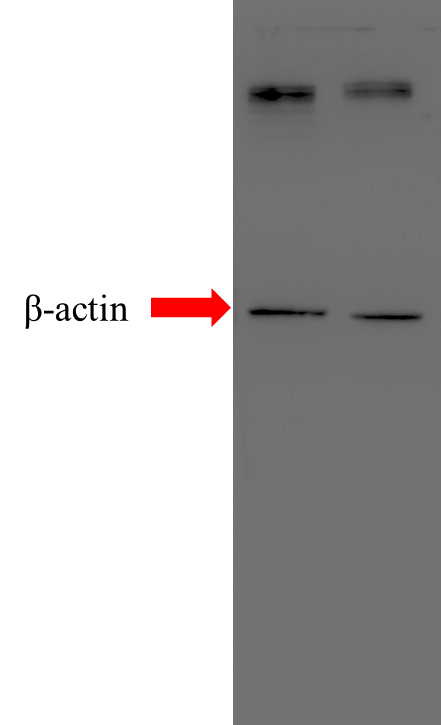


Supplementary Table

Sequence-specific primers for quantitative real-time PCR

Gene Forward primer Reverse primer

CFH 5′-CGTGAATGTGGTGCAGATGGG-3′ 5′-AGAATTTCCACACATCGTGGCT-3′

CFI 5′-TTCCACTGGGTGTTCGTGAC-3′ 5′-TAAAGGCACACTCCGCCAAA-3′

DAF 5′-ACGGTACGTCATCCAACGAG-3′ 5′-AGCCAACGAAGAGTTACGAAGA-3′

Crry 5′-CCAAACAATGTGGGGATAGCAG-3′ 5′-TGTCTGCCAAAGTGGGCTTA-3′

C3aR 5′-TAACCAGATGAGCACCACCA-3′ 5′-TGTGAATGTTGTGTGCATGG-3′

C5aR 5′-GATGCCACCGCCTGTATAGT-3′ 5′-ACGAAGGATGGAATGGTGAG-3′

CFH; Complement factor H, CFI; complement factor I, DAF; decay-accelerating factor, Crry; complement receptor 1-related gene/protein y, C3aR; C3a receptor, C5aR; C5a receptor.
